# Dengue fever and chikungunya virus infections: identification in travelers in Uganda – 2017

**DOI:** 10.1186/s40794-019-0099-3

**Published:** 2019-11-29

**Authors:** John T. Kayiwa, Annet M. Nankya, Irene Ataliba, Charity A. Nassuna, Isaac E. Omara, Jeffrey W. Koehler, John M. Dye, Eric C. Mossel, Julius J. Lutwama

**Affiliations:** 1 Department of Arbovirology, Emerging and Re-emerging Infectious Diseases, Uganda Virus Research Institute, Entebbe, Uganda; 2 US Army Medical Research Institute of Infectious Diseases, Fort Detrick, MD USA; 3 Division of Vector-Borne Diseases, Centers for Disease Control and Prevention, Fort Collins, CO USA

**Keywords:** Dengue, Chikungunya, International traveler

## Abstract

Arboviruses are (re-) emerging viruses that cause significant morbidity globally. Clinical manifestations usually consist of a non-specific febrile illness that may be accompanied by rash, arthralgia and arthritis and/or with neurological or hemorrhagic syndromes. The broad range of differential diagnoses of other infectious and non-infectious etiologies presents a challenge for clinicians. While knowledge of the geographic distribution of pathogens and the current epidemiological situation, incubation periods, exposure risk factors and vaccination history can help guide the diagnostic approach, the non-specific and variable clinical presentation can delay final diagnosis. This case report summarizes the laboratory-based findings of three travel-related cases of arbovirus infections in Uganda. These include a patient from Bangladesh with chikungunya virus infection and two cases of dengue fever from Ethiopia. Early detection of travel-imported cases by public health laboratories is important to reduce the risk of localized outbreaks of arboviruses such as dengue virus and chikungunya virus. Because of the global public health importance and the continued risk of (re-) emerging arbovirus infections, specific recommendations following diagnosis by clinicians should include obtaining travel histories from persons with arbovirus-compatible illness and include differential diagnoses when appropriate.

## Introduction

Febrile illnesses in travelers have been reported in 20 to 70% of cases returning from tropical and sub-tropical regions, and although associated with low mortality rates, significant morbidity has been observed [1]. Fever is by far the most common reason for seeking medical care in returning international travelers according to international data [2]. However, clinical evaluation of fever can be complex as several factors must be considered such as geographic region, period of incubation, as well as signs and symptoms, in order to detect uncommon infections which may be unfamiliar to clinicians in non-endemic regions. Of the travel-related illnesses, arbovirus infections cause substantial public health burden in tropical and sub-tropical regions, and their geographic distribution continues to expand due to a number of complex and interrelated factors such as urbanization, climate change, changes in land-use, spread by viremic travelers and vector range expansion [3].

Dengue virus (DENV), genus *flavivirus*, family *Flaviviridae*, and chikungunya virus (CHIKV), genus *alphavirus*, family *Togaviridae*, are transmitted to humans via bite from the *Aedes (Ae.) aegypti* and *Ae. albopictus* mosquito. Dengue fever (DF) and chikungunya are typically characterized by fever, myalgia, arthralgia, rash, with dengue being more likely to cause severe disease including hemorrhagic complications. Globally, the incidence of DF has increased 30-fold in the last 50 years [4], with four different serotypes known to cause infection. Co-circulation of different serotypes has been observed in areas which previously had circulation of a single DENV serotype [5]. Cohort studies of travelers have found high rates of seroprevalence with a lower incidence of clinical infection, suggesting many cases are asymptomatic or mild and therefore not reported [6]. Because the incubation period of DENV is 4 to 7 days (range 3 to 14) [7], symptom appearance in travelers more than 14 days post-travel will unlikely be travel-associated dengue virus infection.

Chikungunya is normally associated with acute diffuse polyarthralgia with recovery usually within weeks. The virus is antigenically and genetically most closely related to onyong-nyong virus (ONNV) and to a lesser extent, Mayaro and Ross River viruses [8]. A number of CHIKV outbreaks have been reported in Africa, the Middle East, India, and Southeast Asia, and may have spread and caused epidemics in the Caribbean and in the United States, and more recently in Europe [9]. CHIKV outbreaks can involve large numbers of human cases and rapid dissemination of the virus. In the Réunion Island, in an epidemic from April 2005 to June 2006, approximately 270,000 cases were reported, representing nearly 40% of the population. *Ae. aegypti* was the principal vector; however, in recent epidemics in Réunion Island and southern India, *Ae. albopictus* has been co-implicated [10]. In Africa, CHIKV is maintained in an enzootic cycle involving nonhuman primates, but in Asia the human-mosquito cycle predominates, possibly including mechanical transmission [11]. Several studies in Uganda, have reported presence of DENV and CHIKV competent transmission vectors [12–14]. Dengue and chikungunya while endemic in Uganda, are increasingly being detected in travelers, and due to their relatively low natural prevalence in Uganda may be undiagnosed if correct travel histories are not obtained [15]. With increased importation, this can in the case of dengue lead to multiple circulating strains (hyperendemicity) thus increasing the risk of secondary infections and severe dengue.

Laboratory diagnosis of arboviral infections at Uganda Virus Research Institute (UVRI) is accomplished by serologic methods, virus isolation, and group- and virus-specific reverse transcription (RT)– polymerase chain reaction (PCR). A typical serologic algorithm involves testing acute-, and whenever possible, convalescent-phase serum specimens for anti-viral immunoglobulin M (IgM), followed by a plaque reduction neutralization test (PRNT) to confirm a presumptive IgM-positive sero-status. Virus isolation and RT-PCR are used with acute-phase specimens (before day 5 post-onset) because duration of viremia is typically 2–4 days. Herein, we report two serologically confirmed infections of DENV in patients traveling from Ethiopia and, one CHIKV positive infection confirmed by virus culture and RT-PCR in a traveler from Bangladesh, over a one-month period.

## Materials and methods

The Department of Arbovirology, Emerging and Re-Emerging Infectious Diseases at UVRI, established arbovirus surveillance in 2013 to obtain clinical public health data about the causes of acute febrile illness (AFI). In the period between September and October 2017, three cases of suspect arbovirus infection in international travelers coming from endemic areas were registered. Serology, RT-PCR, and virus isolation were performed by the arbovirus laboratory at UVRI, Entebbe. Based on the patients’ journeys from endemic areas for arbovirus infections and on the clinical manifestations, serum samples were collected and divided into three aliquots: 1 for IgM ELISA, 1 for virus isolation and 1 for storage and future testing. Heat inactivated sera were tested for IgM antibodies as described [16]. Virus isolation on Vero cell monolayers was attempted on all samples and cell monolayers observed for cytopathic effects (CPE) over 14 days. Viral RNA was extracted from serum and positive culture supernatant using the QIAamp Viral RNA extraction kit according to manufacturer’s instructions (Qiagen, Hilden, Germany). Isolated RNA was tested with the broadly reactive group-specific PCR for flaviviruses, orthobunyaviruses (in part), and alphaviruses [17], using Qiagen One-Step RT-PCR kit according to manufacturer’s protocol, on a BioRad T100 Thermal Cycler (BioRad, Inc., Singapore). Virus-specific real-time RT-PCR was performed using QuantiTect Probe One-Step RT-PCR kit (Qiagen) according to manufacturer’s instructions on a Stratagene Mx 3000 (Stratagene, La Jolla, CA).

Total nucleic acid from the CHIKV CPE positive cell culture sample was sequenced using the SeqPlex WTA Kit (Sigma-Aldrich, St. Louis, MO), the Apollo 324 System (WaferGen Biosystems, Inc., Freemont, CA), and the MiSeq platform (Illumina, San Diego, CA). Quality trimmed reads were filtered against coliphage phi-X174 (NC_001422.1) and the human H19 reference genome (NC_000001 - NC_000024), and the remaining reads were de novo assembled and BLAST analyzed for identification. The trimmed and filtered reads were mapped against the top BLAST-identified sequence (GenBank MF773566) and the initial draft genome to generate a final genome sequence. All sequence analysis used CLC Genomics Workbench (Qiagen). Sequence alignments and phylogenetic trees (Neighbor joining with Jukes-Cantor and 1000 bootstrap replicates) were generated using the final draft sequences generated and all near full length sequences for each organism available in GenBank.

## Results

### Case report 1: ARB01183/Uganda

A 40-year-old male traveling from Bangladesh was admitted with fever and joint pain at Nakasero Hospital on 09 Sep 2017. Date of initial symptom onset of illness was 08 Sep 2017. The patient had no significant past medical history, was on no regular medication, and had received yellow fever specific travel-related vaccination. The patient, whose final destination was the Democratic Republic of Congo (DRC) where he worked, became ill while in transit in Dubai before traveling to Uganda. Once in Uganda and at Nakasero Hospital, a blood sample was collected on 09 Sep 2017 and transported to the arbovirus laboratory at UVRI.

### Case report 2: ARB01227UVRI and ARB01228UVRI

Less detailed information is available for case report 2 involving two sisters who traveled to Ethiopia to visit their father for about 6 weeks. They became ill but got better after a week with treatment in Ethiopia. Their father had been diagnosed with a case of DF 2 months prior to them falling sick. They traveled back to Uganda on 27 Aug 2017. They arrived at the UVRI clinic on 03 Oct 2017, and a blood sample was collected from each person, approximately 5 weeks post-travel. They looked well but requested a DF test to confirm test results performed in Ethiopia.

### Case report testing

A total of 284 serum samples collected in 2017 were tested for yellow virus (YFV), West Nile virus (WNV), DENV1–4, CHIKV and Zika virus (ZIKV) antibodies by IgM capture ELISA [16]. Only the diagnostic testing results for arbovirus infections of the three travel-related cases are shown and summarized in Table 1. All serological testing in case report 1 (ARB01183/Uganda) were negative, while high neutralizing antibodies against DENV2 were found for both sisters that traveled to Ethiopia in case report 2 (ARB01227UVRI and ARB01228UVRI).

**Table 1:** Test results for arboviral infections

Arboviral Test	ARB01183/Uganda	ARB01227UVRI	ARB01228UVRI
IgM ELISA			
YFV	Negative	Negative	Negative
DENV	Negative	**Positive**	**Positive**
ZIKV	Negative	**Positive**	**Positive**
CHIKV	Negative	Negative	Negative
WNV	Negative	**Positive**	**Positive**
PRNT antibody titers			
YFV	ND*	**< 5**	**< 5**
WNV	ND*	**< 5**	**< 5**
DENV1	ND*	**5**	**< 5**
DENV2	ND*	**2560**	**1280**
DENV3	ND*	**< 5**	**< 5**
DENV4	ND*	**< 5**	**< 5**
ZIKV	ND*	**80**	**40**
Virus culture			
	**Positive**	Negative	Negative
RT-PCR			
Group-specific	**Alphavirus positive**	Negative	Negative
Virus-specific	**CHIKV positive**	ND^a^	ND^a^

^a^*ND* Not Done

The boldface in the results table highlights positive results from negative results

**Fig. 1:**
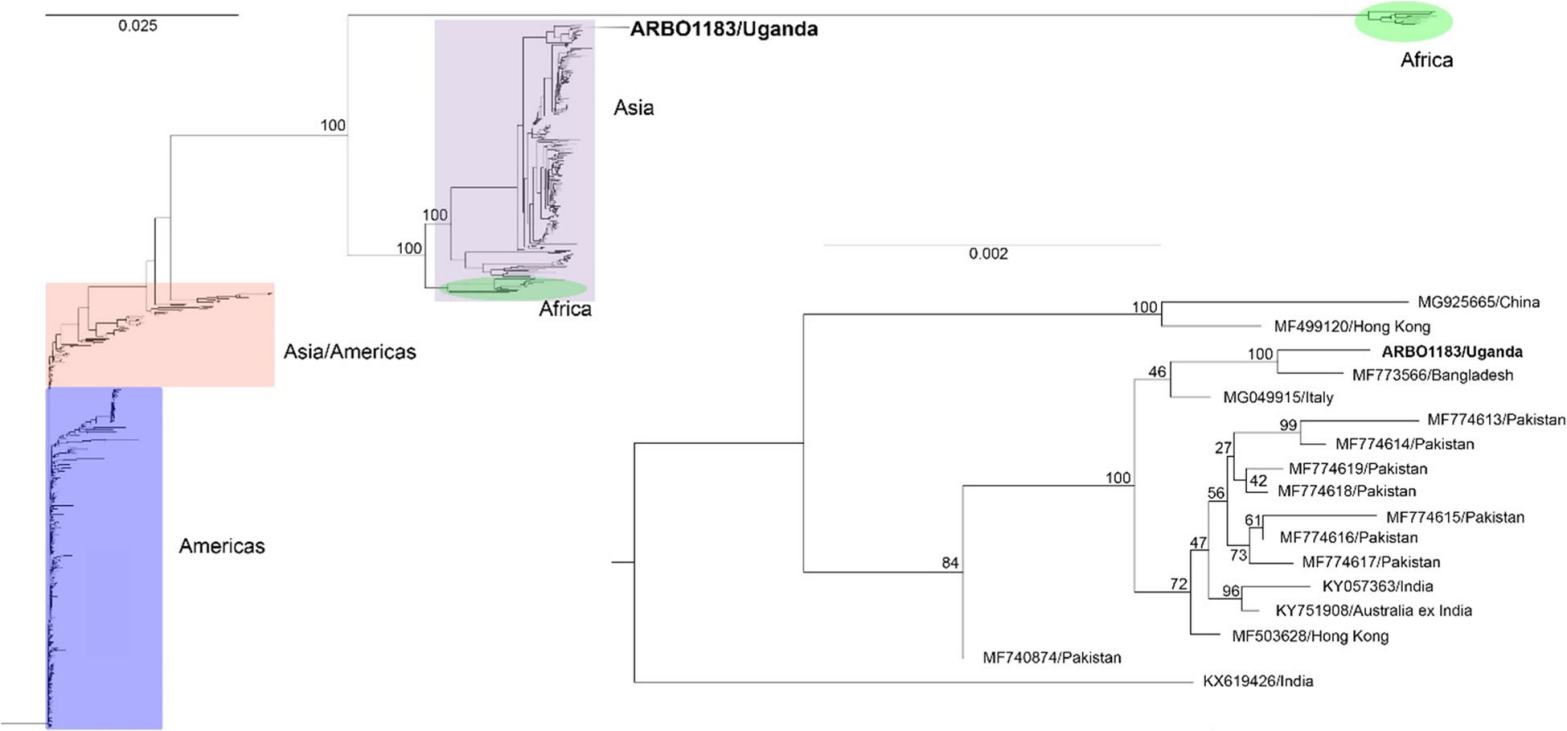
Phylogenetic tree of CHIKV culture isolate ARB01183/Uganda showing the African and Asian genotypes, including the strains that emerged in the Pacific and Brazil

Virus culture on Vero cell monolayers was performed in T25 flasks. The serum sample from the Bangladeshi traveler exhibited prominent and characteristic 100% CPE on day 2 post-infection. RNA extracted from harvested virus supernatant and tested by group-specific RT-PCR was positive for alphavirus and negative for flavivirus and bunyavirus. Further testing by real-time RT-PCR using CHIKV, ONNV and Semliki Forest virus (SFV) specific primers, yielded positive results for CHIKV. Next generation sequencing of the RNA isolated from cell culture supernatant of the CPE-positive culture sample identified a CHIKV genome that genetically clustered within a CHIKV clade containing predominantly Asian CHIKV strains (Fig. 1). The CHIKV strain most related to the virus isolated from ARB01183/Uganda was from Bangladesh collected in 2017 (MF77773566, Fig. 1 insert).

## Discussion

In many malaria-endemic countries, the clinical practice has always been to presumptively treat febrile patients for malaria. However, with improved and easily accessed malaria diagnostics in recent years, a substantial proportion of acutely ill febrile patients in sub-Saharan Africa have been shown not to have malaria [18, 19]. Clinical differentiation of infections caused by alphaviruses and flaviviruses is often not possible in many low- and middle-income countries. Infections by arboviruses circulating in epidemic regions may only be suspected after careful history-taking, and the clinician may be faced with the retrospective interpretation of tests. For case report 1, the positive virus culture on Vero cells at day 2 post-infection was suggestive of an alphavirus infection. The positive group-specific RT-PCR and virus-specific real-time RT-PCR on viral RNA extracted from the culture supernatant confirmed a diagnosis of acute CHIKV infection. No convalescent sample was collected from the patient since a positive PCR result is considered definitive of infection. Three CHIKV genotypes are known; Asia, East/Central/South Africa (ECSA) and West Africa [20]. Phylogenetic inference of the ARB01183/Uganda strain isolated from the patient who travelled to Uganda from Bangladesh showed a relationship to the Asian genotype and was most closely related to the MF773566/Bangladesh strain currently circulating in Asia (Fig. 1).

Case report 2, involving two sisters who traveled to Ethiopia, tested IgM positive for more than one virus (Table 1). Subjects ARB01227UVRI and ARB01228UVRI also had cross neutralizing antibody responses to ZIKV and DENV-2 by PRNT. However, both exhibited DENV-2 neutralization titers significantly greater than the 4-fold determining threshold, suggesting exposure to DENV-2. Extensive IgM cross reactions have been reported in ELISA testing, particularly in patients with previous flavivirus infection or vaccination [21]. This necessitates confirmatory testing of presumptive IgM positive cases by differential PRNT to be included in the routine diagnostic testing algorithm for better result interpretation and patient management, in cases were molecular testing results are negative.

Uganda is a country with environmental conditions conducive to the spread of arboviruses and entomology studies have reported the presence of transmission-competent mosquito vectors [12–14]. The global spread of vectors and importation of new viruses or strains of viruses by infected travelers, would potentially increase rates of endemic transmission. In the case of dengue virus, this means increasing the frequency of multiple circulating strains and thus increased severe dengue [22]. This is illustrated by the recent global spread of CHIKV and ZIKV to previously non-endemic countries [23]. Case report 1 had the potential to introduce an Asian strain of CHIKV to a region that recently reported the spread of the East/Central/South Africa (ECSA) genotype.

## Conclusion

The arbovirus surveillance program at UVRI has the capability to detect, identify and report both endemic and travel-associated arbovirus infections. The rise in incidence of mosquito-borne infections in international travelers highlights the portability of these diseases and the ease with which they can rapidly spread around the world. Therefore, specific recommendations following diagnosis of imported arbovirus infections by clinicians, should include obtaining travel histories from persons with arbovirus-compatible illness and include differential diagnoses when appropriate. Data collected during routine surveillance of febrile illness and associated trends, improve health outcomes and the ability to detect and respond to unusual events of potential public health consequences [24].

Comprehensive and integrated disease surveillance and laboratory systems can generate information used to minimize inefficiencies of disease-specific silos and assure that limited public health resources are leveraged appropriately to improve health security. Also, such systems can identify and detect epidemic-prone illnesses whether existing or novel.

The criteria for arbovirus surveillance and diagnosis must therefore be based on extensive clinical, epidemiological and laboratory findings. Laboratory confirmation is as important in order to distinguish infections with similar clinical manifestations such as dengue fever and other arboviruses. Most low- and middle-income countries do not have systematic processes for collecting, analyzing and putting these data to use, and as such reliable routine data has been identified as one of the main challenges for effective public health action at country level [24]. To bridge this gap, all members of the United Nations (UN) to which Uganda is a member, must be committed to the goals of *The 2030 Agenda for Sustainable Development* [24]. Implementation of such surveillance systems would require continuous technical assistance and training and increased and sustainable funding from both local and international partners.

## Data Availability

The data used and/or analyzed during the current study is available from the corresponding author on reasonable request.

## Abbreviations

CHIKV: Chikungunya virus
CPE: Cytopathic effect
DENV: Dengue virus
DF: Dengue fever
ELISA: Enzyme linked immunosorbent assay
IgM: Immunoglobulin M
PRNT: Plaque Reduction Neutralization Test
RT-PCR: Reverse-transcriptase polymerase chain reaction
UVRI: Uganda Virus Research Institute
WNV: West Nile virus
YFV: Yellow fever virus
ZIKV: Zika virus
